# Short and mid-term outcomes and functional results in metal-on-metal hip resurfacing arthroplasty at 5 years follow-up: the Spanish experience

**DOI:** 10.1186/s12891-019-2498-z

**Published:** 2019-04-16

**Authors:** Olga S. Pérez-Moro, Marcos E. Fernández-Cuadros, Inmaculada Neira-Borrajo, Eduvigis Aranda-Izquierdo, María J. Albaladejo-Florin, Rafael Llopis-Miró

**Affiliations:** 1 Physical Medicine and Rehabilitation Department, Hospital Universitario Santa Cristina, Calle del Maestro Vives 2 y 3, Madrid, Spain; 2 Traumatology and Orthopedic Surgery Department, Hospital Universitario Santa Cristina, Calle del Maestro Vives 2 y 3, Madrid, Spain

**Keywords:** Osteoarthritis, Birmingham hip resurfacing, Hip prosthesis, Survival analysis

## Abstract

**Background:**

Hip resurfacing arthroplasty (HRA) and in particular, Birmingham hip resurfacing (BHR), is commonly employed as an alternative to total hip arthroplasty (THA) in young patients, as it allows for preservation of femoral bone stock and resumption of physical activity. The aim of our study was to investigate 5-year survival and functional outcomes of BHR arthroplasty in young Spanish osteoarthritis (OA) patients.

**Methods:**

This is an observational, prospective, cohort study of patients who underwent BHR between June 2005 and December 2009 at a Spanish public hospital with a minimum follow-up of 5 years. All surgeries were performed by a single surgeon (RLM). Survival was analyzed using the Kaplan-Meier method. Functional outcomes and return to work and physical activities were also assessed.

**Results:**

Five-year survival rate of the prosthesis was 95.74% (95% CI: 95.77–98.07), and estimated 10-year survival was 92.92% (95% CI: 85.07–96.72). Harris hip score significantly increased from 41.13 to 97.63 (*p* < 0.001) at 5-year follow-up. Average time for returning to work and sporting activities was 3.89 (SD: 2.39) and 3.47 (SD: 1.18) months respectively. Failure occurred in 14 patients, 8 of whom experienced femoral neck fractures.

**Conclusions:**

Our data support the short and mid-term efficacy of BHR arthroplasty in young OA patients, indicating good implant survival, improvement in patients’ functionality and a swift return to work and physical activities after surgery.

## Background

Total hip arthroplasty (THA) is regarded as an effective alternative in the treatment of patients with hip osteoarthritis (OA) [1]. Despite being a successful procedure, several studies have indicated that THA may not be the best option in younger patients, due to the higher rate of aseptic loosening and subsequent increased risk of revision surgery observed in this population [1–4]. It has been hypothesized that the higher functional demands and intensity of physical activity, leading to higher wear and mobilization of the prosthesis, may be responsible for the lower success of this procedure in younger patients [5].

Based on this, in the late 70s (1979), hip resurfacing arthroplasty (HRA) attracted surgeons’ attention as a bone conserving alternative to THA for the treatment of young patients with OA [3].

In comparison with THA, HRA does not involve femoral head removal, allowing for the preservation of the proximal femoral bone stock while providing a functional range of motion (ROM), restoring proprioception, hip joint biomechanics and hip stability [6–8]. The first generation of HRA prostheses was, however, associated with a high rate of wear, aseptic loosening (often secondary to osteolysis) and complications such as femoral neck fracture [7–9].

Changes in the design and biomaterials employed, led to the development of a second generation of resurfacing prostheses [10]. This, along with improvements in the surgical technique, had a direct impact on implant wear, endurance and clinical outcomes [10].

A wide variety of bearing materials are currently available for HRA; bearing couples may consist of metal-on-metal, metal-on-polyethylene, ceramic-on-ceramic or ceramic-on-polyethylene systems. The most commonly used bearing couple is the Birmingham Hip Resurfacing (BHR) system, a metal-on-metal (MoM) prosthesis whose components are produced from a highly polished cobalt-chromium alloy (2 μm surface roughness) which results in low wear and friction rates.

This prosthesis has been associated with several advantages. In particular, the use of BHR prostheses with larger diameter heads (36-54 mm) has been shown to reduce the risk of hip dislocation and to provide a greater ROM compared to classic THA [11]. Additionally, BHR has been shown to facilitate femoral replacement and conversion to THA, if needed [11]. On the other hand, the lack of modularity of the implant, which may limit the correction of limb asymmetry, the potential risk of early fractures of the femoral neck (0.4%), adverse local tissue reaction (ALTR) to metal debris or pseudotumor formation represent potential sources of concern with regard to these implants [10, 12–16]. In addition, the long learning curve and the technical complexity of the surgical procedure still limit the use of BHR among surgeons [10, 14]. Despite this, reports showing high short to mid-term survival rates of this prosthesis [17, 18] have rekindled interest in the use of BHR for the treatment of OA in patients with high functional demands.

In light of the above, the objective of this study was to investigate short and mid-term survival of BHR prostheses as well as functional outcomes, in Spanish patients undergoing HRA at a Spanish public hospital.

## Methods

### Study design

A prospective observational study was conducted in 145 consecutive patients who received metal-on-metal BHR arthroplasties between June 2005 and December 2009 at a Spanish public hospital (Hospital Universitario Santa Cristina, Madrid). The study was approved by the Clinical Research Ethics Committee of La Princesa University Teaching Hospital and was performed in accordance with the guidelines of the Helsinki Declaration. All patients provided written informed consent preoperatively. Patients were followed-up for a minimum of 5 years, until October 2015.

### Inclusion/exclusion criteria

The following inclusion criteria were used: women ≤60 years and men ≤65 years presenting with either pain secondary to OA, avascular necrosis (AVN) or developmental hip dysplasia; inability to perform activities of daily living (ADL); asymmetry of the limbs < 3 cm; absence of kidney disease and good bone quality. Women of reproductive age, as well as patients with one or more of the following conditions were excluded from the study: osteoporosis, active rheumatoid arthritis, ankylosing spondylitis, alcoholism, steroidal treatment, renal insufficiency and allergy to metals (Co and Cr).

### Surgical procedure

After a thorough explanation of the advantages and disadvantages of the technique and the differences with classical THA, all of the patients provided informed consent before undergoing BHR surgery. All surgeries were performed with a BHR system (Smith and Nephew) by a single surgeon (R. Llopis-Miró). A modified posterior lateral approach was used in order to preserve the blood supply to the femoral head [19]. In patients in which a posterior approach was deemed inappropriate, an anterior approach was used. All patients received epidural anesthesia. Patients received 2 g of cefazolin as antibiotic prophylaxis before surgery and then every 8 h during the first day. Enoxaparin 40 mg (daily for up to 30 days) and physical therapy (compressive socks plus plantar air pumps) as deep venous thrombosis (DVT) prophylaxis immediately after surgery.

In accordance with the fast track protocol implemented in the hospital since 2005, on the first day after surgery all patients underwent drainage and were mobilized (sitting and standing) for a few seconds. Over the next few days, patients were asked to walk with a cane, learn to transfer (from bed to chair and back again), exercise quadriceps and hamstring muscles, climb stairs and learn to wash and dress unassisted. In patients with signs of either inflammation or bone/tissue damage at follow-up, a biochemical (monitoring of cobalt-chromium ion levels) and clinical evaluation was conducted, to rule out the presence of any ALTR and/or pseudotumors formation.

### Main variables and outcomes

**Fig. 1:**
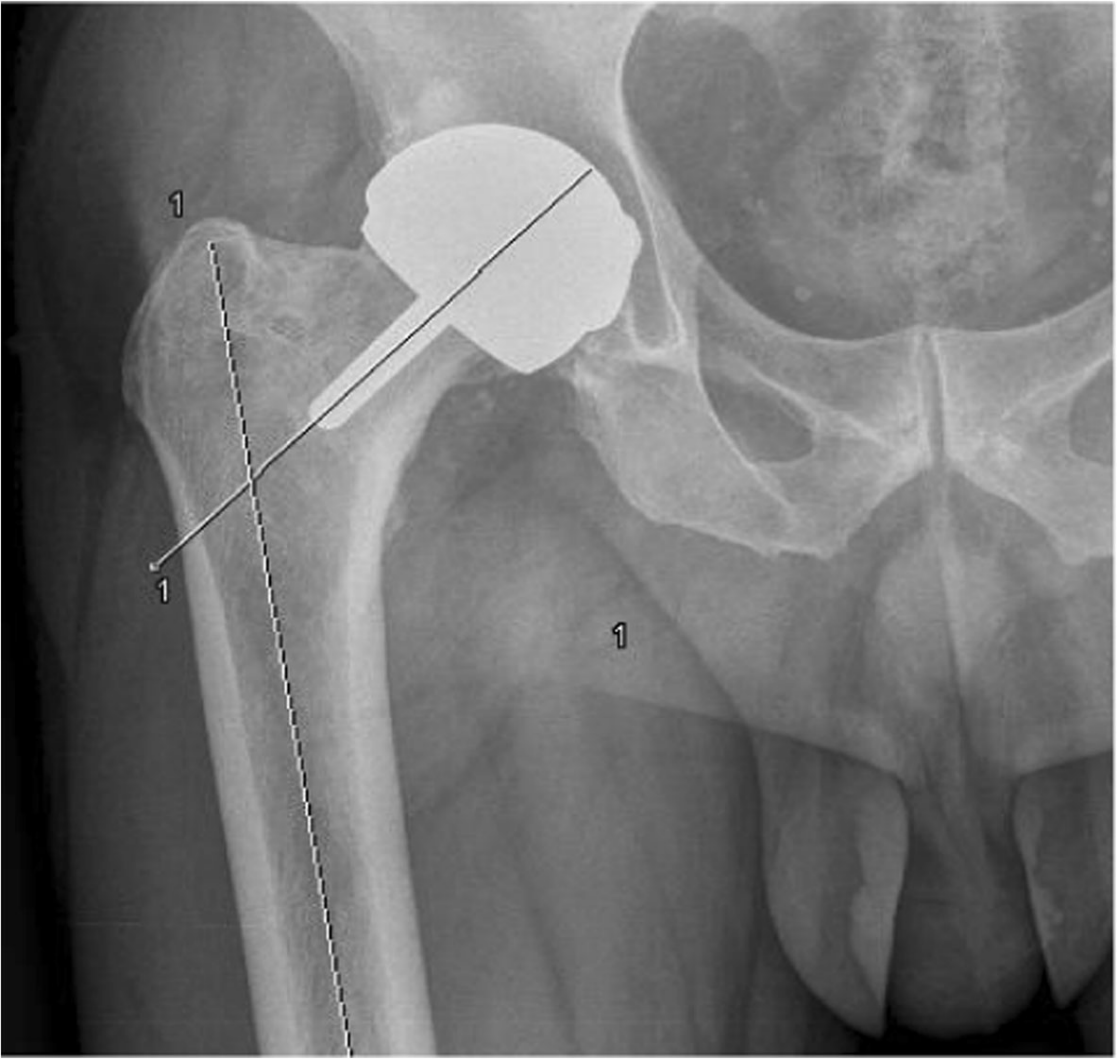
Representative image of the measurement of cervical-diaphyseal angle

Demographic variables analyzed included age, sex, weight, height, body mass index (BMI), ROM and asymmetry of the limbs. Surgical variables included anesthetic type, size of the head and acetabulum of the prosthesis, surgical approach and need for blood transfusion. Radiological variables included femoral offset, acetabular and cervical-diaphyseal angles (Fig. 1). Presence of radiolucencies and neck thinning was also evaluated by radiographic examination.

The primary outcome was the survival of the BHR prosthesis at a minimum follow-up of 5 years (a 10-year-survival rate was also estimated based on the proportion of patients who had undergone a follow-up visit at 10-years). Other variables included length of stay (LOS), need to perform outdoor rehabilitation, and time needed to return to work and to sport activities. Functional outcomes included Oxford hip score (OHS) and Harris hip score (HHS) pre- and post-surgery [20, 21]. Variables for which 5 year follow up data were not available, were analyzed at the maximum follow-up period available.

### Statistical analysis

For quantitative variables, mean, standard deviation (SD), median, minimum and maximum were calculated. For qualitative variables, absolute and relative frequencies were determined. Confidence interval was set at 95% (*p* < 0.05). McNemar, U-Mann Whitney and Wilcoxon tests were used to compare qualitative and quantitative variables where appropriate. Measurements of the same variable from the same sample at different times were compared using the McNemar and the non-parametric Wilcoxon test for qualitative and quantitative variables, respectively. To compare quantitative variables in two different groups of independent patients, the Mann-Whitney U test was used.

Survival of the BHR prosthesis was defined as the time from implantation to the occurrence of any complication during the follow-up period. The survival analysis was carried out using the Kaplan-Meier Method. Implant survival for each patient was computed as the difference between date of operation and date of revision surgery. The Cox-regression method was used to identify predictive factors of prosthesis survival; age, sex, BMI and practice of sports were included in the regression as independent variables.

All statistical analyses were carried out using the STATA® (version 14) statistical package. The level of significance was established at 95% (*p* < 0.05).

## Results

### Demographic, clinical and surgical variables

Over the study period, 145 out of 230 patients completed the minimum 5-year follow-up and were included in the analysis (Table 1). Of these, 117 (80.69%) were male. The mean age of the patients was 49.5 years (SD: 9.67), with a mean BMI of 26.8 kg/m^2^ (SD: 3.54). The most common pre-operative diagnosis was hip OA, accounting for 60% of the surgeries (*N* = 87). With respect to laterality, 53.1% of the surgeries (*n* = 77) involved the right side. Nearly all of the procedures (*n* = 140; 96.55%) were performed using a posterior lateral approach, while an anterior lateral approach was employed for the remaining prostheses (*n* = 5, 3.45%). Mean femoral head size was 50 and 42 mm for male and female patients, respectively. Mean acetabular size was 56 mm for men and 48 mm for women. The number of patients with limb asymmetry significantly decreased from 90 (60%) to 64 (39.9%, *p* < 0.01) after the procedure. Similarly, mean asymmetry showed a significant reduction, going from 0.86 cm (SD: 0.44) to 0.55 cm (SD: 0.38; *p* = 0.001) after the surgery (Table 2). Additionally, hip ROM significantly improved, increasing from 87.90° before surgery to 98.56° 1 year after surgery (*p* < 0.001) (Table 3). At radiological evaluation, mean femoral offset was 2.99 mm (SD: 0.57), mean cervical-diaphyseal angle was 139.28° (SD: 5.6) while mean acetabular angle was 40.3 ° (SD: 7.27) (Table 1). Only 9 patients (6.21%) required a blood transfusion after the surgery (Table 1).

**Table 1:** Baseline characteristics of the study cohort and surgical variables analyzed. (Table should be included in the “Demographic, clinical and surgical variables” paragraph, within Results section)

Variables	
Pre-surgical	
Age (year; SD)	49.52 (9.67)
Male (n; %)	117 (80.69)
BMI (kg/m^2^; SD)	26.8 (3.54)
Surgical	
Hip OA (n; %)	87 (60.00)
Developmental hip dysplasia (n; %)	43 (29.65)
Aseptic bone necrosis (n; %)	12 (8.28)
Legg-Calvé-Perthes disease (n; %)	2 (1.38)
Traumatic hip dislocation (n; %)	1 (0.69)
Right sided (n; %)	77 (53.1)
Left sided (n; %)	68 (46.9)
Posterior lateral approach (n; %)	140 (96.55)
Anterior lateral approach (n; %)	5 (3.45)
Male femoral size (mm; SD)	50
Female femoral size (mm; SD)	42
Male acetabular size (mm; SD)	56
Female acetabular size (mm; SD)	48
Global femoral size (mm; SD)	47.49 (3.57)
Global acetabular size (mm; SD)	53.52 (3.52)
Cervical-diaphyseal angle (°; SD)	139.28 (5.69)
Acetabular angle (°; SD)	40.3 (7.27)
Femoral off-set (cm; SD)	2.99 (0.57)
Need for blood transfusion (n; %)	9 (6.21)
Post-surgical	
Length of stay (days; SD)	3.96 (0.8)
Inpatient Rehabilitation (days; SD)	3.52 (0.7)
Return to playing sports (months; SD)	3.47 (1.8)
Return to work (months; SD)	3.89 (2.39)

**Table 2:** Limb asymmetry pre-surgery and 3-months post-surgery

Asymmetry	Mean	SD	CI 95%	Median	Min	Max	n	Missing data	*p*-value
Pre-surgery	0.86	0.44	0.77–0.95	0.8	0.5	2.5	90	55	0.001
3-months post-surgery (cm)	0.55	0.38	0.46–0.65	0.5	0	2	64	81	Ref.

The *p*-value corresponds to a comparison between the asymmetry values pre-surgery and post-surgery using the Wilcoxon Test

*SD* standard deviation, *CI* confidence interval

**Table 3:** Flexion range of motion (ROM) pre-surgery and post-surgery

Flexion	Mean	SD	CI 95%	Median	Min	Max	n	Missing data	*p*-value
Pre-surgery	87.90	8.09	86.57–89.23	90	40	110	145	0	
1-month post-surgery	86.36	9.25	84,83–87.89	90	0	105	143	2	0.1694
3-months post-surgery	91.92	9.60	90.33–93.51	90	0	110	143	2	< 0.001
6-months post-surgery	96.39	10.13	94.72–98.07	95	0	115	143	2	< 0.001
1-year post-surgery	98.56	10.17	96.88–100.24	100	0	115	143	2	< 0.001

The *p*-value corresponds to a comparison between the flexion ROM values pre-surgery and different post-surgery evaluations using the Wilcoxon Test

*SD* standard deviation, *CI* confidence interval

### Outcome variables

**Fig. 2:**
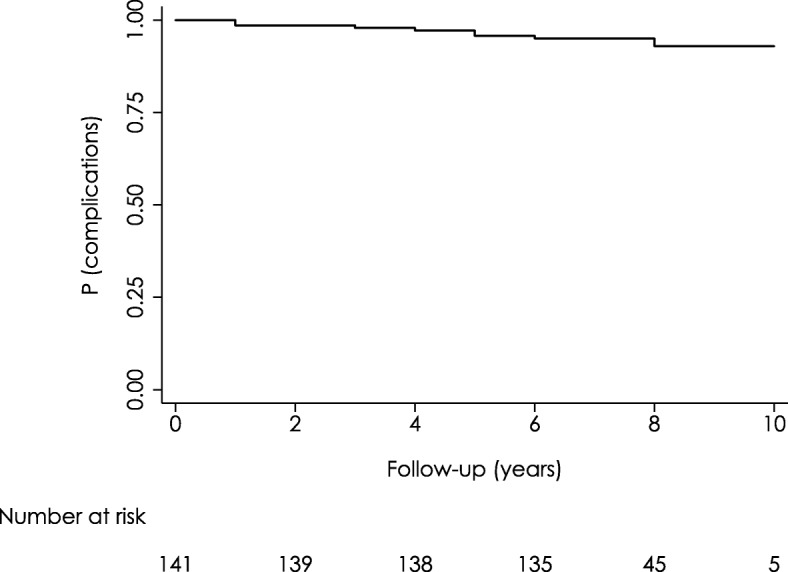
BHR arthroplasty survival evaluated by Kaplan-Meier Method (0–10 years)

Prosthesis survival rate, defined as the time from surgery to the occurrence of any complication, was estimated at 95.74% (CI 95.77–98.07) at 5-year follow-up. In patients for whom 10-year follow-up data were available, the 10-year survival rate was estimated at 92.92% (CI 85.07–96.72) (Fig. 2). When analyzing possible predictors of prosthesis failure, we found that higher BMI was associated with a significantly higher risk of failure (HR: 1.37; *p* = 0.008). Conversely, a significantly lower risk of prosthesis failure was observed in patients who were active in sports compared to patients who were not (HR: 0.13; *p* = 0.069). With regard to gender and age, none of these factors had a significant effect on prosthesis survival.

Mean LOS was 3.96 days (SD: 0.8), with a mean LOS of inpatient rehabilitation of 3.52 days (SD: 0.7). The average time for returning to physical activity was 3.47 months (SD: 1.8) while average time to return to work was 3.89 months (SD: 2.39) after surgery (Table 1).

### Functional outcomes

**Fig. 3:**
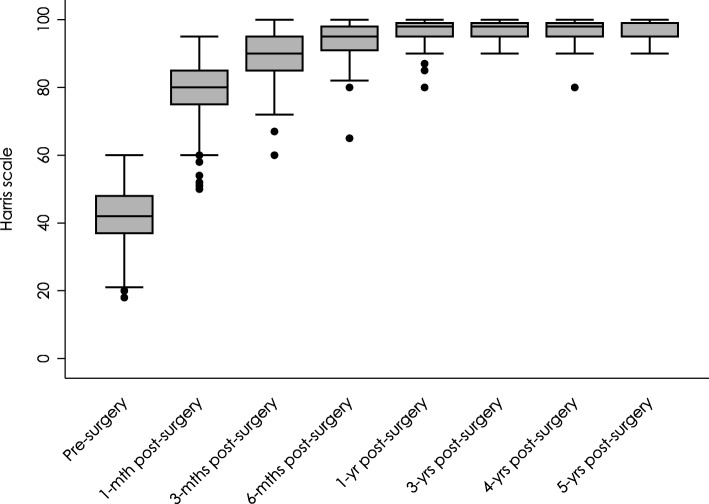
Harris Hip Scores at 0–5 years follow-up

**Fig. 4:**
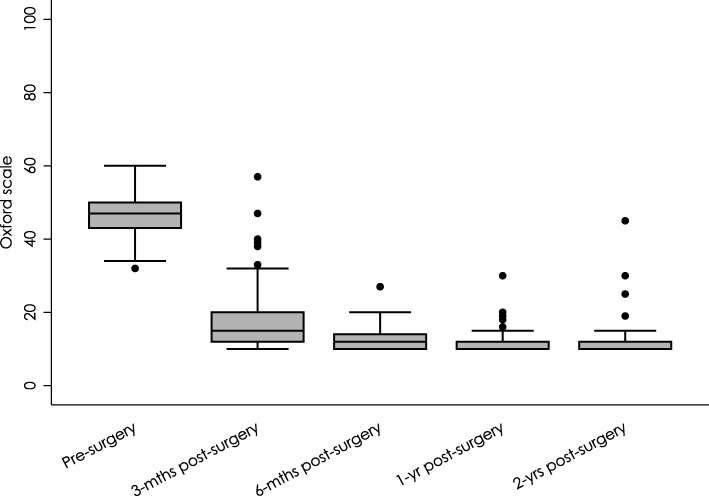
Oxford Hip Scores at 0–2 years follow-up

Average Harris Hip Score (HHS) increased from 41.13 (SD: 9.98) preoperatively to 78.66 (SD: 8.21, *p* < 0.001) 1 month after surgery and progressively improved up to 97.63 (SD: 2.58, p < 0.001) at 5-year follow-up (Table 4, Fig. 3). Similarly, OHS scores indicated that functional capacity significantly improved in the first month after surgery, decreasing from 46.74 (SD: 5.37) to 17.32 (SD: 7.15; p < 0.001). At 2 years of follow-up, the average OHS score was 11.38 (SD: 3.93), indicating a further improvement in functional capacity (Table 5, Fig. 4).

**Table 4:** Harris Hip Score pre-surgery and post-surgery

Harris Hip Score	Mean	SD	CI 95%	Median	Min	Max	n	Missing data	*p*-value
Pre-surgery	41.11	9.83	39.50–42.72	42	18	60	145	0	
1-month post-surgery	78.66	8.21	77.31–80.02	80	50	95	143	2	< 0.001
3-months post-surgery	88.99	6.33	87.94–90.03	90	60	100	143	2	< 0.001
6-months post-surgery	88.99	6.33	87.94–90.03	90	60	100	143	2	< 0.001
1-year post-surgery	96.77	3.68	96.16–97.38	98	80	100	142	3	< 0.001
2-years post-surgery	97.18	3.37	96.61–97.74	99	80	100	137	8	< 0.001
3-years post-surgery	97.55	2.42	97.15–97.96	98	90	100	137	8	< 0.001
4-years post-surgery	97.28	3.05	96.76–97.80	98	80	100	134	11	< 0.001
5-years post-surgery	97.63	2.58	97.19–98.07	99	90	100	134	11	< 0.001

The *p*-value corresponds to a comparison between the Harris Hip Scores pre-surgery and post-surgery using the Wilcoxon Test

*SD* standard deviation, CI confidence interval

**Table 5:** Evaluation of Oxford Hip Score pre-surgery and post-surgery

Oxford Hip Score	Mean	SD	CI 95%	Median	Min	Max	n	Missing data	*p*-value
Pre-surgery	46.74	5.37	45.86–47.62	47	32	60	145	0	
3-months post-surgery	17.32	7.15	16.14–18.50	15	10	57	143	2	< 0.001
6-months post-surgery	12.83	2.91	12.34–13.31	12	10	27	143	2	< 0.001
1-year post-surgery	11.41	2.49	11.00–11.82	10	10	30	143	2	< 0.001
2-years post-surgery	11.38	3.93	10.70–12.06	10	10	45	143	2	< 0.001

The *p*-value corresponds to a comparison between the Oxford Hip Scores pre-surgery and post-surgery using the Wilcoxon Test

*SD* standard deviation, *CI* confidence interval

### Complications

**Fig. 5:**
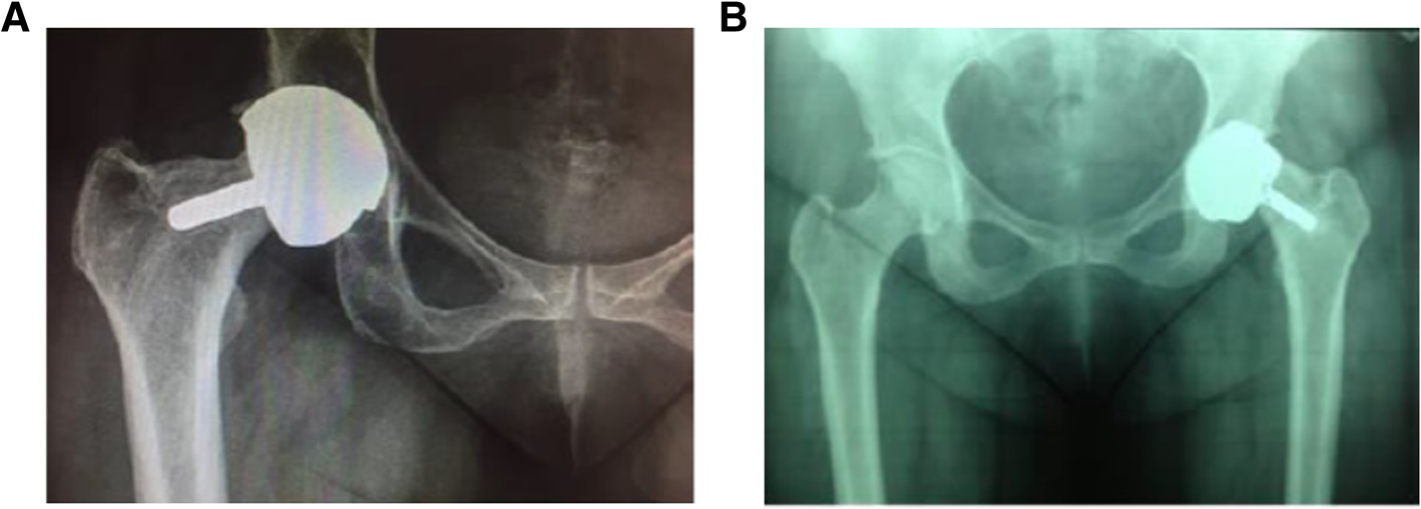
Radiographic images showing evidence of neck thinning in two patients: case 1 (**a**) and case 2 (**b**)

**Fig. 6:**
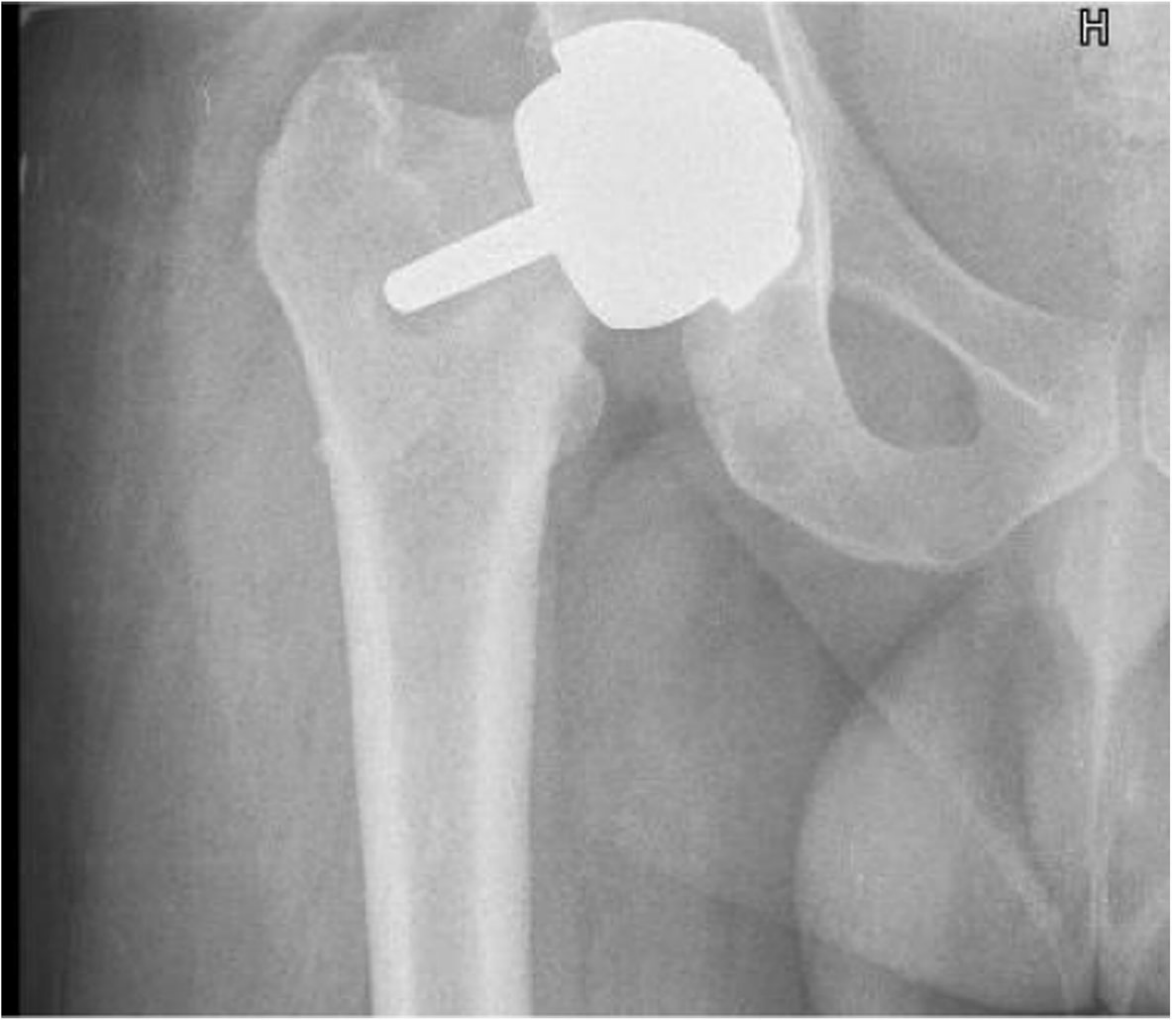
Radiographic image showing radiolucency in one patient

During the follow up period, there were 8 fractures (5.52%), 2 collapses of the femoral head (neck thinning) (1.38%), 2 revision surgeries secondary to pain (1.38%), and a psoas tendonitis (0.69%) (Table 6, Fig. 5). Finally, evidence of radiolucency indicative of osteolysis was described in one patient (0.69%) (Fig. 6). No patients showed ALTR or pseudotumor formation during follow-up.

**Table 6:** Complications observed during the follow-up period

Complication	n (%)
Fractures (n; %)	8 (5.52)
Collapses (neck thinning) (n; %)	2 (1.38)
Revision surgery (n; %)	2 (1.38)
Osteolysis (n; %)	1 (0.69)
Psoas Tendoinitis (n; %)	1 (0.69)

All fractures occurred between 2 and 3 weeks after surgery and none of them was secondary to a fall. At revision surgery, the femoral component was replaced with a metal-on-metal Synergy stem with a large femoral head (Smith & Nephew). The psoas tendonitis resolved after steroidal infiltration. One of the 8 patients reporting fractures suffered from wound infection secondary to *Enterobacter cloacae* that resolved with a specific antibiotic treatment. No vascular or nerve lesions, dislocation or heterotopic ossification were reported in the study cohort.

## Discussion

To the best of our knowledge, this is the first study to report short- to mid-term clinical outcomes of HRA using BHR performed by a single surgeon in a Spanish Public Hospital. Our findings indicate that BHR may be an effective treatment option for young patients with OA, with a 5-year survival rate of 95.74% and a predicted 10-year survival rate of 92.92%. The relevance of this report lies in that, although limited to a single center, it provides information regarding BHR’s effectiveness in routine clinical practice in Spain, allowing for benchmarking both nationally and internationally, thus laying the basis for further improvement of the quality of care for patients with OA in our setting. The efficacy of BHR in terms of prosthesis survival have been previously evaluated in several other studies in different settings [17, 22, 23]. In particular, in a study conducted in England in young patients (mean age 52.1 years) with OA, Treacy and McBryde reported 5-year and 6.1-year survival rates of 98 and 93.2%, respectively [22]. The similarities between these results and the one reported in the present study may be due to the similar characteristics of the studies’ cohorts (i.e. young active patients) and the surgical approach chosen. In a longer follow-up study conducted in a similar population, Murray et al. found that survival of BHR prostheses was strongly influenced by gender, approaching a 95% survival rate at 10 years in male patients vs. only 74% in female patients [23]. Data from Daniel et al. also support the mid- to long- term efficacy of BHR arthroplasty in patients with high functional demands, with 10-year and 15-year survival rates of 97.4 and 95.8%, respectively [24]. Similarly to what was observed by Treacy et al., a significantly lower survival rate was observed in women (91.5%) compared to male patients (98.0%) at 15 years follow-up [24]. In contrast, we observed no significant impact of female gender on prosthesis survival. This may be explained by the fact that, given the smaller sample size and higher proportion of women in the study cohort, this study may not have been powered enough to detect such differences.

It has been proposed that BHR prosthesis may allow the resumption of an active lifestyle, addressing the expectations of young, active patients with OA who would like to return to a normal level of physical activity after the surgery. Despite this, only a few studies have investigated return to sporting activities following BHR arthroplasty [25]. In our study, we observed a mean return to physical activity after approximately 3.5 months, demonstrating that young patients treated with BHR may resume preoperative sporting activity soon after surgery. These results are in line with those reported by Sandiford et al. [25], who observed that young patients with OA (mean age 55.8 years) who underwent BHR arthroplasty were able to return to sports by 3 months and perform the same number of activities at preoperative intensity.

Given the encouraging results achieved through BHR arthroplasty, mainly due to advances in the implant design and surgical approaches as well as to a better understanding of hip biomechanics and implant placement, the popularity of BHR has increased over the last decade [25]. Nevertheless, despite its many theoretical advantages (i.e., bone stock preservation, ease of revision surgery and reduced stress transfer due to a large femoral head), the use of BHR has been associated with complications such as avascular necrosis (AVN), periprosthetic femoral neck fracture and pseudotumor formation [15, 16, 26, 27]. Periprosthetic femoral neck fracture generally occurs within the first 3 months after surgery and reports from the literature suggest a prevalence of approximately 1 to 4% in patients who receive a BHR prosthesis [26–29]. It is noteworthy to mention that Quesada et al. have reported that a high rate of periprosthetic fracture is generally observed in the first 50 hip surgeries (*n* = 11; 22%) of a surgeon’s learning curve, and progressively diminishes to one fracture (2%) in the next 50 surgeries and no fractures at all in the next 200 surgeries [30]. In our study, the incidence of femoral neck fractures was slightly higher than the one reported in the literature, with 5.52% of the study patients reporting this complication in the follow up period. This may be partly explained by the high average BMI of the study population (26.8 kg/m2). Indeed, elevated BMI has been described as a potential risk factor for periprosthetic femoral neck fracture [31]. Other factors include poor bone stock quality, improper surgical technique, excessive varus of the implants, neck femoral notch, necrosis of sub-capital head and femoral head cysts > 2 cm [31, 32].

AVN of the remaining proximal femur has also been reported to predispose to periprosthetic femoral neck fracture [14]. Reaming of the femoral neck and avascular injury due to the surgical approach have been proposed as possible mechanisms for AVN after resurfacing [33–37]. In our series, two AVN of the femoral head (1.38%) were reported over the 5-year follow-up period. Although the true incidence of AVN is not known [36], our results are close to those reported by Daniel et al., and De Smet et al., who observed 0.2 and 0.4% rates of AVN, respectively [17, 38].

Pseudotumor formation is also recognized as a serious complication and a potential risk factor for HRA failure in patients undergoing this procedure [15, 16]. Pseudotumors usually develop as solid or fluid masses in the peri-prosthetic soft tissue, as a reaction to metal debris; however, their pathogenesis has not yet been clarified [39]. Interestingly, changes in the head-neck ratio and femoral neck thinning have been associated with pseudotumor formation in patients undergoing metal-on-metal HRA [40]. In our study, two patients (1.38%) showed neck thinning at radiographic examination; however, no ALTR or pseudotumor formation was observed during follow-up. Aseptic loosening of femoral and acetabular components and prosthesis dislocation have also been described as possible complications of both HRA and THA [1–4, 7–9]. In our study, only one case (0.69%) of aseptic loosening and no dislocations were observed over the 5-year follow-up period, suggesting a low risk of these complications in patients receiving a BHR.

This study must be understood in the context of its limitations. Mainly, the observational design of the study may predispose to selection bias. Nevertheless, it allows for an evaluation of the prosthesis efficacy under routine clinical conditions, thus adding to the external validity and generalizability of the study results. Another possible limitation may be related to the reduced availability of data regarding long-term prosthesis survival (≥10 year) in the study cohort. Despite this, studies from the literature have reported similar long-term survival rates as the one observed in this study [17, 22, 23], thus suggesting that our estimation of BHR 10-year survival may be representative of the intended target population.

## Conclusion

Overall, our results indicate that besides showing good implant survival, BHR arthroplasty provides a significant improvement in patients’ functional status and allows a prompt return to both work and physical activities. Based on this, BHR may be regarded as an effective alternative in the treatment of young active patients with hip OA in Spain. Nevertheless, further investigation and perhaps the creation of a national arthroplasty registry may help confirm these findings in the general population.

## Abbreviations

ADL: Activities of daily living
ALTR: Adverse local tissue reaction
AVN: Avascular necrosis
BMI: Body mass index
CI: Confidence interval
HHS: Harris hip score
HRA: Hip resurfacing arthroplasty
LOS: Length of stay
MoM: Metal on metal
OA: Osteoarthritis
OHS: Oxford hip score
ROM: Range of motion
SD: Standard deviation
THA: Total hip arthroplasty
